# *Vital Signs:* Trends in Human Rabies Deaths and Exposures — United States, 1938–2018

**DOI:** 10.15585/mmwr.mm6823e1

**Published:** 2019-06-14

**Authors:** Emily G. Pieracci, Christine M. Pearson, Ryan M. Wallace, Jesse D. Blanton, Erin R. Whitehouse, Xiaoyue Ma, Kendra Stauffer, Richard B. Chipman, Victoria Olson

**Affiliations:** ^1^Division of High-Consequence Pathogens and Pathology, National Center for Emerging and Zoonotic Infectious Diseases, CDC; ^2^Epidemic Intelligence Service, CDC; ^3^Division of Global Migration and Quarantine, National Center for Emerging and Zoonotic Infectious Diseases, CDC; ^4^National Rabies Management Program, Wildlife Services, Animal and Plant Health Inspection Service, U.S. Department of Agriculture, Washington, D.C.

## Abstract

**Introduction:**

Each year, rabies causes approximately 59,000 deaths worldwide, including approximately two deaths in the United States. Before 1960, dogs were a common reservoir of rabies in the United States; however, increasingly, species of wildlife (e.g., bats, raccoons) are the main reservoirs. This report characterizes human rabies deaths, summarizes trends in rabies mortality, and highlights current rabies risks in the United States.

**Methods:**

Rabies trends in the United States during 1938–2018 were analyzed using national rabies surveillance data. Data from the Healthcare Cost and Utilization Project for 2006–2014 were used to estimate the number of postexposure prophylaxis (PEP) visits per 100,000 persons during 2017–2018. The Centers for Medicare & Medicaid Services’ average sales price data were used to estimate PEP costs.

**Results:**

From 1960 to 2018, a total of 125 human rabies cases were reported in the United States; 36 (28%) were attributed to dog bites during international travel. Among the 89 infections acquired in the United States, 62 (70%) were attributed to bats. In 2018, approximately 55,000 persons sought PEP after contact with a potentially rabid animal.

**Conclusions and Comments:**

In the United States, wildlife rabies, especially in bats, continues to pose a risk to humans. Travelers also might be exposed to canine rabies in countries where the disease is still present; increased awareness of rabies while traveling abroad is needed. Vaccinating pets, avoiding contact with wildlife, and seeking medical care if one is bitten or scratched by an animal are the most effective ways to prevent rabies. Understanding the need for timely administration of PEP to prevent death is critical.

*On June 12, 2019, this report was posted online as an *MMWR *Early Release.*

## Introduction

Rabies virus, a *Lyssavirus* that infects mammals, is transmitted through saliva, most commonly from the bite or scratch of an infected animal. In the United States, several variants, or strains, of rabies virus circulate in animal reservoirs, including raccoons, skunks, foxes, and bats (*1*). Rabies virus infection, regardless of the variant or animal reservoir, is fatal in over 99% of cases, making it one of the world’s most deadly diseases. There is no treatment once signs or symptoms of the disease begin, and the disease is fatal in humans and animals within 1–2 weeks of symptom onset. Prompt administration of postexposure prophylaxis (PEP), consisting of rabies vaccine and immune globulin, immediately after exposure effectively prevents disease (*1*,*2*).

The elimination of canine rabies virus variant (CRVV) from the United States is one of the most important public health successes of the 20th century. However, globally, approximately 59,000 persons still die from rabies every year; 98% of these cases are caused by CRVV (*3*). At the beginning of the 20th century, CRVV was enzootic in the United States, but beginning in 1947, animal vaccination and leash control laws led to improved rabies control nationwide. Canine rabies and associated human rabies cases fell sharply (*4*). By the late 1960s, fewer than 500 rabid dogs and three human rabies cases were reported annually (*5*).

In the United States, CRVV was eventually eliminated in 2004 (*6*) through use of parenteral and oral rabies vaccines. As the prevalence of CRVV declined, rabies viruses associated with wildlife reservoirs such as skunks, foxes, raccoons, and bats accounted for an increasing proportion of cases in animals and humans in the United States. Wildlife rabies is found in all states except Hawaii (*1*). Since the late 1970s, raccoon rabies has spread across the Eastern Seaboard from Alabama to Maine, causing the largest epizootic of animal rabies in U.S. history (*7*). Given the close proximity of raccoons to residents of suburban neighborhoods and trends toward urbanization, human exposures to rabies increased (*8*,*9*).

The use of oral rabies vaccine, composed of vaccine wrapped in a flavored bait, has been successful in controlling westward spread of raccoon rabies.* However, outside oral rabies vaccination zones, raccoon rabies virus variant accounts for nearly 75% of the terrestrial animal rabies cases reported in the United States (*1*). In areas where both raccoon and bat rabies occur, human rabies exposures are 600% higher than in areas where only bat rabies occurs (*1*,*9*).

Although domestic animal exposures account for a large portion of human PEP usage, bat rabies virus variants are responsible for most human rabies deaths in the United States (*1*). This apparent paradox might be due to several factors, including lack of awareness of the risk of acquiring rabies from bats, or difficulty identifying bat bites and scratches (*10*). This analysis highlights current rabies risks in the United States, and assesses the cost and public health impact of rabies control efforts.

## Methods

U.S. National Rabies Surveillance data maintained by CDC’s Poxvirus and Rabies Branch were analyzed to assess trends in human and animal rabies in the United States during the past 81 years (1938–2018) (*1*). Initial risk assessment and treatment for exposure to a rabid animal commonly occurs in the emergency department because of the need for wound treatment and rabies immune globulin, typically only available in emergency departments (*11*).

The Agency for Healthcare Research and Quality’s Healthcare Cost and Utilization Project’s (HCUP; https://www.hcup-us.ahrq.gov/) 2006–2014 data, which include longitudinal U.S. hospital care data, were used to estimate the rate of PEP visits (number per 100,000 persons) for 2017–2018 based on the U.S. population. HCUP patient data from emergency departments with an *International Classification of Diseases, Ninth Revision* diagnosis code of V04.5 (need for rabies prophylaxis), were evaluated (https://hcupnet.ahrq.gov). In addition, 2017 national sales data for rabies immune globulin were provided by an independent consultant (Marketing Research Bureau, Inc., unpublished data, 2019).

The 2019 Centers for Medicare & Medicaid Services average sales price data were analyzed to estimate the cost of PEP (*12*,*13*). The average sales price data lists rabies immune globulin at $312 per 150-IU dose (a 165-pound [75-kg] adult needs 10 doses and a 95-pound [45-kg] child needs 6 doses) and rabies vaccine at $290 per dose (4 total doses needed). The average PEP cost and range were determined using the 2019 average sales price data and previously published data from 2004, adjusted for inflation (*13*,*14*).

The cost and frequency of U.S. public health system rabies responses were derived from previously published literature and opinions of subject matter experts (*13*,*15*,*16*). An economic analysis conducted by CDC provided estimates of the number of imported dogs from countries at high risk for rabies and the public health cost associated with importation events (*15*).

## Results

**FIGURE 1:**
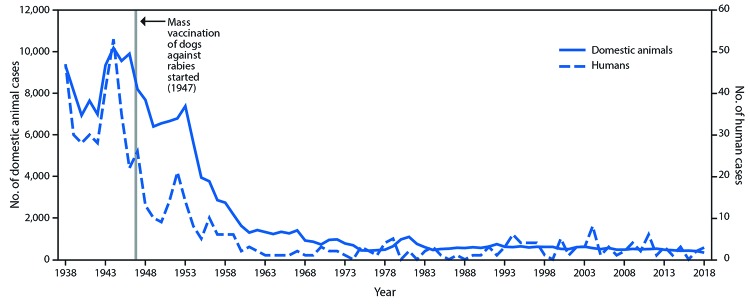
Rabies cases in humans and domestic animals — United States, 1938–2018

**FIGURE 2:**
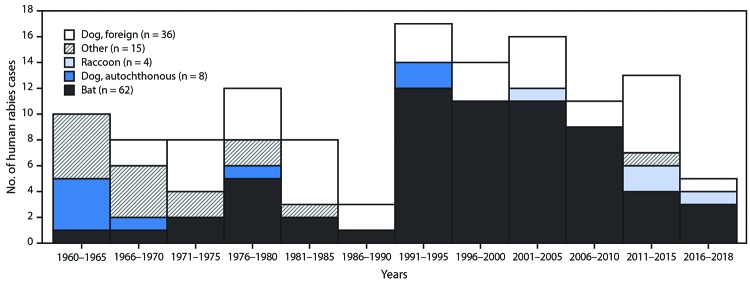
Rabies virus variants* associated with human rabies cases (N = 125)^†^ — United States, 1960–2018 * Other rabies virus variants included skunk, fox, and unknown. ^†^ Includes 120 persons who died and five survivors with suspected rabies infection in 1970, 1977, 2004, 2009, and 2011. Cases in survivors were never laboratory-confirmed; three cases are included in bat rabies virus variants because of epidemiologic links to bats and two are included in other (one unknown and one lab-acquired).

During 1938–2018, 588 cases of human rabies were reported in the United States. The elimination of CRVV in the United States through canine rabies vaccination has resulted in a tenfold decrease in human rabies cases reported from 1938 through 2018 (Figure 1). During 1960–2018, among 125 reported human rabies cases, 89 were U.S.-acquired, including six organ transplantation cases. Among all U.S.-acquired cases, 62 (70%) were caused by bat rabies virus variants (Figure 2). Since 1960, 36 (28%) U.S. residents have died of rabies acquired from dogs while traveling abroad.

During 2017–2018, an average of 55,000 (range = 45,453–66,000) persons were treated for potential rabies exposure each year. The cost for rabies PEP averages $3,800 (range = $1,200–$6,500), not including costs for hospital treatment or wound care. This results in annual estimated PEP costs of $209 million (range = $66 million–$358 million).

Since 2003, the U.S. public health system has responded to approximately two human rabies deaths, 175 mass bat exposure events (events where >10 persons are exposed to a potentially rabid bat), and one rabid dog importation every year (Table). CDC estimates that 1.06 million dogs enter the United States every year, including 107,000 (10%) that are imported from countries where CRVV is enzootic, thereby posing a potential risk for reintroduction of CRVV into the United States. Since 2015, three canine rabies cases have been imported in rescue dogs adopted from countries with a high risk for rabies. Canine rabies importation events are estimated to cost $213,833 (range = $171,066–$256,599) per event in public health response and health care costs to prevent the spread of the disease to humans and their pets. Total estimated costs associated with rabies public health emergency response activities are $7.6 million per year (range = $2.6 million–$12.9 million) (Table).

**TABLE:** Estimated annual costs associated with emergency rabies responses — United States, 2017–2018

Type of rabies response/No. of exposures	Response item	Estimated costs
**Human cases**		
2 cases per year*	Investigation	$42,900 (1,300 hours^†^ at $33 per hour^§^)
x 39 contacts per case^¶^	PEP	+ $148,200 (39 contacts x $3,800 per course**)
Total = 78 exposed contacts	Investigation and PEP	= $191,100 per case
Total cost for human cases		**= $382,200 total cost per year (2 cases)**
**Mass bat exposures^††^**		
3.5 exposures per agency per year	Investigation	$2,871 (87 hours^†^ at $33 per hour^§^)
x 50 state/territorial agencies^§§^	PEP	+ $38,000 (10 persons x $3,800 per course**)
Total = 175 exposures per year	Investigation and PEP	= $40,871 per exposure
Total cost for bat exposures		**= $7,152,425 total cost per year (175 exposures)**
**Rabid dog importation events^¶¶^**		
1 event every 1–2 yrs	Investigation and PEP	$218,833 per 2 years
Total cost for importation events		**= $109,416 total cost per year (1 event)**
**Total annual cost**		**$7,644,041**

**Abbreviation:** PEP = postexposure prophylaxis.

* Annual average of total number of cases reported during 1960–2018.

^†^ Estimated average hours devoted to investigation estimated from information in a previously published report. https://onlinelibrary.wiley.com/doi/full/10.1111/zph.12105.

^§^ Cost per hour derived from 2019 epidemiologist salary listed by Bureau of Labor Statistics. https://www.bls.gov/ooh/life-physical-and-social-science/epidemiologists.htm.

^¶^ Estimated contacts per year were based on previously published data. https://www.intechopen.com/books/non-flavivirus-encephalitis/human-rabies-epidemiology-and-diagnosis.

** Average cost for PEP course determined using 2019 Centers for Medicare & Medicaid average sales price data (https://www.cms.gov/Medicare/Medicare-Fee-for-Service-Part-B-Drugs/McrPartBDrugAvgSalesPrice/2019ASPFiles.html) and previously published 2004 data, adjusted for inflation (https://www.sciencedirect.com/science/article/pii/S0264410X08006373?via%3Dihub). Cost includes immunoglobulin and rabies vaccine; does not include costs for hospital treatment or wound care.

^††^ Mass exposures defined as >10 persons exposed to a potentially rabid bat. Estimated number of exposures per state/territorial agency per year based on previously published data. https://onlinelibrary.wiley.com/doi/full/10.1111/zph.12289.

^§§^ Includes agencies in 49 states and Puerto Rico; Hawaii not included because wildlife rabies is not found in the state.

^¶¶^ Number of importation events and related costs described in previously published report. https://www.federalregister.gov/d/2019-00506.

## Discussion and Conclusions

Bats are currently the leading cause of human rabies deaths in the United States. Unlike rabies management programs targeting raccoon, fox, and coyote populations, bat vaccination is not yet logistically feasible, nor are any rabies vaccines currently approved for use in bats. Despite the rabies exposure risk, the vast majority of bats submitted for testing (94%) do not have rabies (*1*). Thus, widespread killing of bats is not recommended to prevent rabies. However, increased awareness of the risk for rabies from bats and knowledge of when to seek medical attention for PEP are needed. In addition to bat rabies cases, international travel-related rabies cases occur because of a lack of awareness about the ongoing global risk of rabies in dogs.

Efforts to control rabies in wildlife and maintain canine rabies elimination in the United States require ongoing, high-quality rabies surveillance and timely response capabilities. Rabies continues to be a priority zoonotic disease for One Health collaboration (*17*), requiring multi-agency cooperation to ensure continued success of the U.S. rabies control program. Currently, U.S. public health laboratories and United States Department of Agriculture Wildlife Services test approximately 100,000 animals per year, and approximately 5,000 are rabies-positive (*1*). Although CRVV has been eliminated from the United States, dogs might still acquire rabies from wildlife.

Whereas canine rabies vaccination is required throughout the United States, animal registration and rabies vaccination laws vary by county, making it difficult to estimate the current rabies vaccination coverage rates among dogs in the United States. In addition, recent antivaccination sentiments have been documented in owners reluctant to vaccinate their dogs against diseases (*18*). Failure to vaccinate dogs against rabies could constitute a considerable public health threat to both humans and animals. Thus, maintaining current rabies vaccination rates of at least 70% in dogs is critical not only to protect pets, but to protect pet owners as well (*19*).

The findings in this report are subject to three limitations. First, although rabies is a notifiable disease for both humans and animals, data on PEP use among persons seeking care for a potential exposure are limited and rely on emergency department data, some of which may be incomplete. Second, previously published data and current average sales price data from the Centers for Medicare & Medicaid were used to estimate costs for this analysis, but the actual amount hospitals bill for PEP varies considerably, making it difficult to assess the true cost of PEP (*10*). Finally, rabies prevention and control costs have a high degree of variability. For example, costs for public health emergency responses can vary considerably between states depending on the number and type of animals and humans involved.

As the human urban environment encroaches into wildlife settings, human rabies exposures continue to occur. However, the relatively few human rabies deaths that occur in the United States are a testament to the robust response capabilities of the nation’s public health system, as well as the success of wildlife and pet vaccination programs and the availability of effective PEP. Although human rabies is now a rare disease in the United States, it remains one with extremely high consequences.

## Recommendations

A critical component of rabies prevention in the United States is to avoid contact with wildlife, especially bats. Contact with a bat includes bites and scratches, which are often small and can be overlooked. Contact might also occur unknowingly if a bat is present in a room with a young child or mentally impaired person, including a child or person under the influence of medication, drugs, or alcohol or a person who is asleep. In those cases where unrecognized contact might have occurred, persons should assume they have a potential exposure to rabies if the bat is not available for testing and urgently seek care from their medical provider. If the bat can be safely collected and tested, this can inform the need for PEP.

CDC Travelers’ Health provides vaccination recommendations for international travelers (https://www.cdc.gov/travel). Although the risk of travel-associated rabies infection is generally low, travelers should know the risk, avoid contact with animals, have a plan to get care if they are scratched or bitten, and have travel health insurance to pay for treatment should they need it. Travelers at higher risk (i.e., those who might be working with animals abroad or come into close contact with animals while traveling) should additionally consider preexposure prophylaxis vaccination and be aware that PEP is still recommended after a potential exposure, even among vaccinated persons (*2*).

Human rabies is 99% fatal. However, it is 100% preventable through vaccinating pets against rabies, avoiding contact with wildlife and unknown animals, and seeking medical care as soon as possible after being bitten or scratched by an animal.

**Summary**

**What is already known about this topic?**

Each year, rabies causes approximately 59,000 deaths worldwide, including approximately two deaths in the United States. Rabies can be prevented with timely administration of postexposure prophylaxis (PEP).

**What is added by this report?**

During 1960–2018, among 89 U.S. acquired human rabies cases, 62 (70%) were attributed to bats. Dog bites acquired during international travel were the cause of 36 cases.

**What are the implications for public health practice?**

Awareness of the risk of rabies from wildlife, especially bats, and during international travel is needed. Understanding the need for timely administration of PEP to prevent death is critical.

## Footnotes

* https://www.aphis.usda.gov/aphis/ourfocus/wildlifedamage/programs/nrmp/ct_rabies.
